# Experiences of oncology healthcare personnel in international medical service quality: a phenomenological study

**DOI:** 10.1186/s12912-023-01249-1

**Published:** 2023-03-31

**Authors:** Chi-Chun Lai, Shih-Ying Chen, Hsien-Wei Chen, Hsueh-Yu Li, Hsiang-Hao Hsu, Li-Chin Chen, Woung-Ru Tang

**Affiliations:** 1 Department of Ophthalmology, Chang Gung Memorial Hospital, Keelung, Taiwan; 2 International Medical Center, Chang Gung Memorial Hospital, Linkou, Taiwan; 3 School of Nursing, College of Medicine, Chang Gung University, No.259, Wenhua 1st Rd., Guishan Dist, Taoyuan, 33302 Taiwan; 4 Department of Otolaryngology, Chang Gung Memorial Hospital, Linkou, Taiwan; 5 Department of Nephrology, Chang Gung Memorial Hospital, Linkou, Taiwan; 6 Department of Nursing, New Taipei Municipal Tucheng Hospital, New Taipei City, Taiwan; 7 Department of Pediatric Hematology-Oncology, Chang Gung Memorial Hospital, Linkou, Taiwan

**Keywords:** International medical services, Medical tourism, Quality of health care, Hospital oncology service, Qualitative research

## Abstract

**Background:**

With the globalization of medical services on the rise, Asia has ascended to a destination of choice for its high-quality medical services at very reasonable rates. Monitoring the quality of the international medical industry is vital to maintain service demand. The experiences of healthcare personnel (HCP) involved in international medical services (IMS) regarding the provision of services to international cancer patients have not yet been discussed. This study aimed to explore oncology HCP experiences of IMS quality in caring for international cancer patients in Taiwan.

**Methods:**

Descriptive phenomenological method and were analyzed through Colaizzi’s seven-step approach. In this study, 19 respondents were collected data by using in-depth semi-structured interviews. An average interview lasted approximately 45 min.

**Results:**

Four major themes were identified from the interviews: patient selection, psycho-oncology care, predicaments, and promoting suggestions. Additionally, thirteen subthemes emerged, including necessary selection of patients, reasons for unwillingness to enroll international patients, helpless patients, emotional distress, care with warmth, insufficient manpower, an unfair reward mechanism, poor hardware equipment, the predicaments of oncology care, various publicity strategies, one-on-one service model, design of a designated area, and reasonable benefit distribution.

**Conclusions:**

This study explored oncology HCP experiences of IMS quality in caring for international cancer patients, with implications for hospitals in developing high-quality IMS. Due to the fact that IMS is a global trend, HCPs, administrators, and policy-makers are advised to improve the quality of IMS in the oncology department, which has been the least studied field in IMS quality.

## Background

The World Health Organization (WHO) defines international medical service (IMS) as seeking medical services abroad, whereas the World Tourism Organization (WTO) terms the cross-border movement for medical care, disease, and health treatment (or rehabilitation and recuperation) as medical tourism [1, 2]. Medical tourism and IMS are often used interchangeably to refer to the practice of seeking medical services abroad while participating in other tourist activities [3, 4] to the financial benefit not only of the medical institution but also of the host country overall [5–11]. Globalization of medical services is rising, with Asia ascending to a destination of choice due to high-quality medical services at very reasonable rates [4]. Increasing numbers of international patients travel to Asia annually to seek medical services [5, 11], with an estimated 5.3 million international patients traveling in 2017 [12]. As IMS grows rapidly, monitoring IMS quality is vital [8, 11, 13].

Ranking 16th among the top 46 medical tourism destinations [14], Taiwan is a fecund site for the further research and development of high-quality IMS interventions. Nearly 382,000 foreigners came to Taiwan for medical services in 2019, with an increase of 330% from 2009, which created an output value of over $4.5 billion USD [15]. Although Taiwan has met demand, IMS in Taiwan has been insufficiently researched. To date, only three studies have investigated the quality of IMS in Taiwan [16–18]. Two studies explored the perceptions and experiences of medical tourists who sought health examinations and general surgery in Taiwan [16, 18], whereas the other study tested a model for cultivating cultural sensitivity [17]. However, all of the studies only focused on patients’ perceptions and not on the perceptions or experiences of healthcare personnel (HCP) [16–18]. To improve the quality of IMS, researchers should explore this issue from the patient’s viewpoint and from the provider’s perspective.

Outside of Taiwan, previous studies on IMS have mostly focused on questionnaire development and testing [19–22], factors that affect IMS development [23–27], international patients’ selections of destinations [28–30], customer loyalty [31–34], revisiting intentions [35–37], and competitiveness of IMS [4, 38]. Most of the studies were quantitative studies and focused on patients with nonurgent diseases (e.g., dental care or general surgery) seeking short-term treatments (< 4 days) [39, 40]. Moreover, no studies have discussed the quality of IMS in patients with urgent illnesses, such as cancer. Due to the fact that cancer is an important global chronic illness that requires long-term treatment [41], it is crucial to understand HCP opinions on improving IMS quality for cancer patients. Therefore, this study aimed to explore oncology HCP experiences of IMS quality in caring for international cancer patients, with implications for hospitals in developing high-quality IMS.

## Methods

### Study design

This qualitative study was conducted by using a descriptive phenomenological approach, which was previously proposed by Husserl [42, 43]. Descriptive phenomenology is the best choice when the research topic is intended to gain insight into the common features of any lived experience, which can represent the true nature of the phenomenon that is being investigated [43].

### Study participants

Respondents were recruited from the international medical center of the largest medical center in Northern Taiwan, which is part of Asia’s most prominent medical chain system and provides medical services for 20% of Taiwanese cancer patients. HCPs involved in caring for international cancer patients were enrolled in this study. To increase the credibility of the study, a variety of respondents were selected from the volunteers via maximum variation sampling, which is a type of purposeful sampling design [44]. The maximum variation sampling selects participants based on a wide range of variations in backgrounds [44, 45], which encompasses different sexes, ages, positions, and educational levels. A total of 19 HCPs (6 attending physicians, 5 outpatient/ward nurses, 7 case managers, and 1 radiologist) participated in this study without any refusal or dropouts. Table 1 lists the respondent characteristics.

**Table 1:** Respondents’ Characteristics (N = 19)

Code	Gender	Age, y	Occupation	Department
A01	F	42	**Outpatient Nurse**	Radiology
A02	F	30	**Outpatient Nurse**	Radiology
A03	F	53	**Outpatient Nurse**	Radiology
A04	F	37	Case manager	IMC
A05	F	43	Case manager	IMC
A06	F	45	Case manager	IMC
A07	F	32	Case manager	IMC
A08	F	30	Case manager	IMC
A09	F	28	Case manager	IMC
A10	M	57	Attending physician	Radiology
A11	M	60	Attending physician	Oncology
A12	F	41	Radiologist	Radiology
A13	M	53	Attending physician	Radiology
A14	M	59	Attending physician	Gynecology
A15	F	30	Case manager	IMC
A16	M	56	Attending physician	Oncology
A17	F	32	**Ward Nurse**	GS
A18	M	58	Attending physician	Oncology
A19	F	44	**Ward Nurse**	Oncology

Abbreviations: F, female; M, male; IMC, international medical center; GS, general surgery

### Data collection

During spring 2018, The PI (WRT), who was a female professor with a PhD degree in nursing, conducted individual, in-depth, face-to-face interviews in a quiet place that was chosen based on the respondents’ preferences for the interview. A semi-structured interview guide was developed based on literature reviews and discussions with experts who have experiences in IMS. The PI introduced herself and provided a brief introduction about the purpose of the study to evoke respondents’ interests and to establish the relationship. The PI supplemented the interview guide (Table 2) with open-ended questions to gain a deeper insight into the points of view of the HCP. According to descriptive phenomenology, researchers should set aside or bracket their prior experiences in the clinical environment, preconceived notions, or assumptions during the research to reduce subjective bias [42, 43]. The interview was audio recorded with the consent of the respondents. An average interview lasted approximately 45 min, and a research assistant (SYC) obtained demographic data via structured questionnaires after the interview.

**Table 2:** Semi-structured Interview Guide

Questions	
1.	Describe your experience in performing international medical services, especially in the development of international medical care in hospitals?
2.	In your opinion, what are the factors that promote and obstruct the development of international medical care in hospitals?
3.	How would you assess the quality of the international medical services currently offered in the hospital?
4.	What recommendations do you have for the improvement of the quality of international medical services?
5.	What advice do you have for breaking bad news and discussing treatment options with international medical patients, especially those from China and Southeast Asian countries?
6.	What is the cultural difference, which is very important in international medical care, that we should pay more attention to during the process of providing care?
7.	Is there anything that was not included in this interview, but is essential to the topic discussed today that you would like to share with me?

### Data analysis

Qualitative research design entails simultaneous data collection and analysis, and enrollment is terminated when data saturation is obtained [46]. The recorded interviews were transcribed verbatim by the research assistant within 24 h of the interview. The PI (WRT) and a nursing PhD student (SYC) who were trained in qualitative research then jointly analyzed the data via Colaizzi’s (1978) seven-step approach [47], which has been enlightened by Morrow et al. and Shosha [43, 48]. Microsoft Excel 2019 (Taipei, Taiwan) was used for data management. The seven-step approach was performed as follows: (1) Familiarization: each transcript was repeatedly read several times to obtain a general sense about the entire content. (2) Identifying significant statements: after reading each transcript, significant statements related to the phenomenon were identified. Statements were recorded on a separate sheet that noted their pages and line numbers. (3) Formulating meanings: meanings that were relevant to the phenomenon were formulated from these significant statements. After obtaining these meanings, the researchers examined their relevance to the initial statements to ensure their correctness. (4) Clustering themes: the formulated meanings were categorized into subthemes and themes based on their similarity. (5) Developing an exhaustive description: the findings of the study were integrated into a comprehensive description of the phenomenon by incorporating all of the themes. (6) Producing the fundamental structure: the comprehensive description was condensed into a short, dense statement. (7) Seeking verification of the fundamental structure: the findings were returned to participants to ensure whether they captured their experiences. Any relevant new feedback that emerged during this process was incorporated into the results of the study [43, 48].

### Rigor and trustworthiness

The trustworthiness of this qualitative study was evaluated by using Lincoln and Guba’s four proposed criteria [49]: credibility, transferability, dependability, and confirmability. Two authors jointly analyzed the interview data and further discussed and clarified the process to achieve the desired consistency. Maximum variation sampling allowed for the inclusion of respondents with various characteristics, and this methodology strengthened the overall credibility of the study. This also aided in the collection of diverse perspectives to increase transferability. In the case of vague statements, the meanings expressed by the respondents were subsequently clarified and reconfirmed. Additionally, we performed participant checking in an IMS meeting to confirm the credibility and applicability of the results. All of the participants confirmed that the results resonated with their experiences, and no new feedback had developed in their practice since the initial interviews. Although we included respondents with various characteristics, most of the participants shared consistent perspectives and no divergent or conflicting perspectives. The researchers retained documents relevant to the interview data collection and analysis process to establish an audit trail to improve research dependability and confirmability.

### Ethical issues/statement

This study was accepted by the Institutional Review Broad of the institution (No. 201800232B0C602). The principal investigator (PI, WRT) explained the purpose and methodology of the study to the respondents before proceeding with the study. Respondents were also allowed to withdraw from the study at any time, and their participation or refusal to participate did not affect their employment in any way. The respondents’ names were replaced with codes. Moreover, any private information was kept confidential and not made publicly available. All of the obtained data were used solely for academic purposes. The in-depth one-on-one interviews were conducted only after consent forms were signed by the respondents.

## Results

Most of the international patients in the study site are Chinese individuals from various countries who come to seek medical treatment. They mainly communicate in Chinese, and there is no communication barrier. Additionally, most international patients are from high socioeconomic classes and do not trust medical treatments in their home country or are dignitaries who do not intend to expose their diseases to others. Therefore, these individuals attach great importance to the timeliness and privileges of medical treatments. Additionally, most of the participants expressed that the majority of international cancer patients who come to the hospital for treatment are already in the advanced stages of cancer. Their tolerance to the side effects of treatments is generally low, which often leads to the interruption and failure to successfully complete the plan. Therefore, the therapeutic effect is affected. Most international patients listened to many professional treatment suggestions before seeking medical treatments abroad. Due to the fact that they trust the medical standards of the study site, as well as the fact that the medical fees are reasonable, they chose to come to the study site for medical treatments. Furthermore, they came to Taiwan to seek much faster, cutting-edge, and high-end curative cancer treatments, as opposed to those who only go to Taiwan for health check-ups. HCPs need to pay extra attention to care, as international cancer patients have greater medical and service requirements than general patients.

### Identified themes

Four major themes and thirteen subthemes emerged from the lived experiences of the participants (Table 3). The corresponding sample sentences are presented below.

**Table 3:** Major themes and sub-themes of IMS quality in the oncology department

Major themes	Sub-themes
Patient selection	Necessary to select patients
	Reasons for unwillingness to enroll international patients
**Psycho-oncological care**	**Helpless patients**
	**Emotional distress**
	**Care with warmth**
**Predicaments**	**Insufficient manpower**
	**An unfair reward mechanism**
	**Poor hardware equipment**
	**The predicaments of oncology care**
**Promoting factors**	**Various publicity strategies**
	**One-on-one service model**
	**Design of a designated area**
	**Reasonable benefit distribution**

### Patient selection

Due to the differences in disease types and treatment methods, some patients are suitable to come to Taiwan to receive IMS, whereas some patients with advanced cancer are unsuitable for treatment abroad. Therefore, most physicians are unwilling to treat such patients. Physicians will communicate with patients based on professional judgment. If necessary, physicians will dissuade such patients from coming to Taiwan for treatment.

Due to the fact that the participants cared for international patients suffering from cancer with disease characteristics that are different from those of general diseases, they suggested that it was necessary to screen eligible patients to avoid causing burden for both patients and HCPs. For example, Participant 18 noted:

*“Actually, in terms of IMS, it is still necessary to screen eligible patients to a certain extent. We may not accept patients with complicated conditions since we do not have sufficient wards and manpower for such patients.”*

The participants specifically explained the suitable types of international patients and the reason why cancer patients are not suitable. For example, Participant 14 stated:

“*For example, it’s much simpler to perform orthopedic surgery! A patient undergoing orthopedic surgery can return to their country after resting for a week following the surgery. Therefore, one-time medical treatment is the most suitable type. However, cancer patients are more special. Some cancer patients come to Taiwan to undergo surgery, and they still need subsequent chemotherapy. For such patients, I will advise them to return to their countries to receive chemotherapy since the drugs are almost the same…”*.

In addition, Participant 11 provided a further explanation: “*Whether patients are suitable to receive international medical services is subject to the nature of the disease. For example, patients who need to receive one-time treatment or corrective surgery for congenital disability only have to come to Taiwan once a year, and their treatments can be ended in two weeks. Therefore, it’s OK! For example, plastic surgery is very simple, Da Vinci surgery can be completed in a single visit, and patients can return home within 1–2 weeks after the surgery. These treatments are totally fine for international patients. However, the disease types of the Division of Oncology require continuous treatments for a period of time. In addition, it is also necessary to monitor the disease progress and side effects, which is not easy.”* Furthermore, disease prognosis is also one of the factors affecting physicians’ screenings of patients. If the disease progression of a patient is poor, physicians will usually dissuade the patient from coming to Taiwan for treatment. For example, Participant 18 stated: “*We also need to evaluate the patients’ conditions in terms of international medical services. If I believe that the condition of a patient may be worsened within a very short period of time and the therapeutic effect is limited, I won’t ask the patient to come to Taiwan.*”

Apparently, although medical and nursing education has repeatedly emphasized the importance of fair care for all patients, international cancer patients are not treated as local patients. There are many unknown reasons underlying this scenario. Moreover, these factors can only be deeply understood through in-depth interviews. Due to the fact that most of the IMS oncology patients coming to the study site to receive treatments are those who with advanced cancer, their disease may progress rapidly, and their disease condition may be complicated and require long-term monitoring. Furthermore, international patients must be cared for by the attending physician without the assistance of a resident physician. As a result, some attending physicians will not actively receive international patients unless they are forced to. Participant 11 candidly stated:

*“Unless the patient specifically requests to be treated by me, I will not actively receive such patients. I will only provide medical treatment when patients truly need me; otherwise, I won’t actively receive such patients because I feel that I can only provide them with limited assistance. I strongly suspect how we can help such patients if they truly intend to receive treatments in Taiwan.”*

Participant 11 even provided an example by stating: “*Previously, there was a patient who kept emphasizing his privilege since he came to Taiwan, but he didn’t gain any benefit from it at all! What were the problems? First, his previous treatments in Mainland China were unclear, as were his medical records. Based on our judgments, the cause of his severe bleeding was not simply proton therapy but the unusual radiotherapy he previously received in Beijing, which led to local vascular rupture. Under such a circumstance, if we received this patient, we had to bear all responsibilities!”*

Participant 11 also stated: “*Cancer requires repeated treatments for a long period of time (1–3 years), and cancer patients have to receive treatment at least once to twice per month. Therefore, how can international cancer patients maintain such treatments? In addition, cancer patients need to receive not only treatments but also monitoring of side effects. Therefore, it is necessary to consider the long-term benefits instead of the short-term benefits. If an international patient abruptly seeks medical treatment from our ER and I am requested to care for this patient without assistance (from a resident physician) (international patients need to be cared for by the attending physician in person), to be honest, I am already exhausted by caring for local cancer patients…”*.

Participant 11 further indicated: *“I feel embarrassed to reject the patients who come to Taiwan to seek medical treatment from me. Since such patients specifically ask for me to provide them with medical services, I have to at least check their medical condition and tactfully tell them that they should stay in Taiwan only when it is necessary. However, so far, none of the international patients who asked for me stayed in Taiwan. Advanced cancer cannot be treated by a single surgery but requires repeated treatments. Most patients may experience progression within five or six months. If they keep coming to and leaving Taiwan, the treatments cannot be maintained. In addition, how can I treat such patients if they experience side effects and progression when they are not in Taiwan?”*

Furthermore, Participant 18 also clearly stated: *“I truly hate to see international cancer patients pass away here because they truly can’t go home and rest in peace. If I believe that a patient cannot obtain any benefit by coming to Taiwan and may even experience an unexpected situation in a short period of time based on my experiences, I’ll persuade them to receive treatment in their own country. I will directly tell the patient that the physical burdens, expenses, and uncertainties are all too high due to the repeated round trips. I will recommend that the patient receive treatment in their own country because I don’t want to give them an unrealistic hope.”*

### Psycho-oncological care

During the process of cancer treatment, international patients and their family members will inevitably experience emotional distress. The exploration of the causes of such distress and the HCP’s efforts to meet the emotional needs of international patients through communication and treatment is referred to as psycho-oncological care. Although most international patients are from wealthy families with a better social support system, the purpose of these patients with advanced cancer coming to Taiwan for medical treatment is to seek a potentially final hopeful treatment. Therefore, even for international patients who receive proton therapy at the outpatient clinic and no need to be hospitalized, HCPs should also pay attention to the psychological care needs of such patients. After all, in an unfamiliar country and medical system, patients require adaptations and assistance in seeking medical treatments and daily life essentials (including food, housing, and transportation), thus accentuating the importance of psycho-oncological care. Participant 12 provided a vivid example about a child with leukemia by saying:

“International patients are quite helpless. For a hospital that intends to improve IMS quality, in addition to the medical profession, I think that psychological care is very important as well. Because I’ve seen many parents taking their children to come to Taiwan because no effective treatment is available in their home country. Coming to Taiwan is their last glimpse of hope. The mother of the sick child truly knelt outside the clinic and asked the physician to treat her son because this was the last hope for him. Sometimes, we reflect on the level of holistic care and find that the psychological support for cancer patients and their family members is truly very important.”

Most international cancer patients seek potentially final hopeful treatments in Taiwan. Compared with the patients who are declared to be incurable in their home country or other countries (in addition to their home country, some patients have also received IMS in the U.S. and Japan), patients who can receive IMS still have an opportunity to be treated, which provides hope and joy to these individuals. However, such patients usually have worse conditions, and the discomforting symptoms accompanied by cancer and treatments tend to induce emotional distress in patients. Therefore, small unsatisfaction may progress into extreme unhappiness and cause significant issues. For example, Participant 16 said:

“Some trivial things may have a significant impact on the patients (patients may maximize their dissatisfaction). These critically ill patients come to Taiwan in a happy mood because they know that they can receive care. However, under the circumstance of physical discomfort, they will maximize their feeling and dissatisfaction…”

As a result, participants gave a reminder that HCPs must pay attention to psychological needs of international patients during treatments in the hospital. When patients return to their rental accommodation after experiencing a day of treatment (the international patient receiving proton therapy at the outpatient clinic will typically stay in Taiwan for approximately 1 month; thus, they will rent a place to stay near the hospital to facilitate the daily round trips for treatments), it may be the starting point of the pain. Participant 13 emotionally stated: “*These patients (those who come to Taiwan for proton therapy) actually receive daily treatment in the hospital for only about an hour. Therefore, the rest of the time (23 hours) is when they are most vulnerable and need assistance in life.*”

Regarding how to provide psycho-oncological care, the participants indicated the importance of empathy and communication. Participant 16 stated: “*I will empathize with some of their ideas. I will tell them: ‘You have made a lot of effort and homework. The books you are reading are at the same level as those I read during my study in medical school. However, I studied in medical school for seven years, but you have to read these books in three or six months. You are truly working hard!’ Many patients cried when they heard what I said!”*

Due to 45% of the international patients at the study site are from mainland China, their interaction model between HCPs and patients is significantly different from that at the study site, thus creating a significant contrast. Participant 12 said: “*The patients all feel that our medical and nursing personnel are more friendly because they were shouted at by medical and nursing personnel at the hospital in their country. Our HCP is willing to spend two hours developing a good relationship with children (children with cancer) in pre-preparation (pre-positioning of proton therapy). A mother was once touched by our HCP and almost cried because she said that even she was not as patient as the HCP at our hospital for her child.”*

Participants attributed the HCP’s attitude of care with warmth to medical education in Taiwan. Participant 18 stated: “*What are the advantages of our international medical care? The advantage lies in medical education in Taiwan, which educates us to care more about patients and treat them in a humane manner. I believe that this is the significant difference between our care and foreign care.*” As for the timing for providing psycho-oncological care, it is necessary to provide patients with such care at the very beginning of the treatment (instead of at the end stage of treatment). In particular, if a good trust relationship can be developed with pediatric cancer patients, patients’ emotional distress can be reduced, and their adherence can be further increased. Participant 10 said:

*“No matter how advanced the treatment equipment is, it is still cold and definitely will be very stressful for patients. Therefore, I think that psychological interventions should not be provided at the end but should be provided at the very beginning, especially for children. For example, if a 3- to 4-year-old child is not nervous and is highly cooperative for all examinations and treatments, there is no need to anesthetize the patient. Patients typically have to receive proton therapy 30 times, which means that these children have to undergo anesthesia 30 times and experience physical suffering.”*

Participant 10 even further shared his findings during the internship at MD Anderson Cancer Center in the U.S. by stating: “*I saw a psychologist accompanying a pediatric caner patient to come to the outpatient clinic. This is very necessary and important because patients may even save money to be spent on anesthesia. More importantly, the risk for patients can be reduced because every anesthesia poses a risk.*” As a result, Participant 10 aggressively intends to promote psycho-oncological care. He said, “*I intend to promote psycho-social care because I’d like to know whether care and appeasement are what patients and their family members need. I believe that it is very important to understand it. However, I have no idea whether we have actually met the needs of patients.*”

### Predicament

Although the study site has been satisfactorily recognized by most of the patients, during the process of providing international medical services, there are still many hardware and software deficiencies existed in the study site, which may have a negative impact on high-quality IMS. As a result, there is still room for improvement. Based on the experiences of the participants, the current difficulties in IMS at the study site include insufficient manpower, an unfair reward mechanism, poor hardware equipment, and the predicaments of oncology care.

Although high-end medical technology (e.g., proton therapy) in Taiwan has attracted many international patients to seek medical treatment, there are still many difficulties in our hospital that hinder the IMS development of the oncology department. For example, the lack of manpower is a major predicament for IMS development. Almost all of the participants indicated that most international patients expect to receive full-time care from their dedicated HCP. However, with limited manpower and an overwhelming number of international patients, HCPs are unable to meet these patient needs. In fact, they could only devote a limited time for each patient, which may decrease the service quality and patient satisfaction. For instance, Participants 10 and 13 stated the following:

“When you don’t have enough manpower, you can never provide optimal service, no matter how good you claim the service quality is. In times of stress, no one can maintain a calm temperament toward patients, even those with the highest EQ [Emotional Intelligence Quotient]. Only through adequate manpower can we ensure the quality of customer service. After all, this is a service by people.”

“With the case manager constantly running around, some instructions are overlooked in the process. Consequently, we, the attending physicians, have to mention it again, but we don’t dare to tell the case manager to come back and be more attentive because she just brought in 3 international patients to the clinic. It would not be right to do so. Yes, it’s a manpower issue. Some may suggest that the people work harder, but I don’t think that it’s possible.”

The service requirements for international patients are higher than those for general patients. Additionally, they tended to ask for timely responses to their needs. Therefore, HCPs need to exert more effort to coordinate and arrange their treatments and examination appointments. The HCP’s assistance sometimes extends to the patient’s daily life in Taiwan; therefore, the HCP will spend their off-hours working without due compensation. Participants believed that, with regards to the conditions of insufficient manpower and unlimited work loadings, a reasonable reward system was essential. However, the rewards provided by hospitals for IMS are not proportional to the efforts. Participant 7 noted:

“When I told the nurses in the clinic, they were all in disbelief. ‘What? You do all that work for USD 30?’ Do you know how long we spend on our patients? Patients add us in communication applications (e.g., Line, WeChat, and WhatsApp) so they can contact us instantly. They send us messages while we are off from work, on holiday, or even late at night, and we still have to reply to them. When we choose to reply to nonurgent messages the next morning, patients complain to the doctor or supervisor and say that we ignored them and were very slow to respond. Sometimes, we would like to let them know that we are undercompensated. We also want to do it well, but have to respect the limit of our service and protect ourselves from exploitation.”

Moreover, this lack of a reasonable reward system may reduce the morale of the staff and affect their service quality. The following statement was mentioned by Participant 16:

“I have heard that there is a special ‘overtime’ fee given to the staff, but it is a very small reward, so I would rather not take it [this means that this monetary reward to the nurse does not compensate for the amount of effort required for taking care of international patients, so some nurses would rather decline the assignment].”

In addition to the manpower and rewards system issues, the hard equipment of the institutions is another essential component for IMS, which is also the basis of the first impression of international patients who arrives to the hospital. This scenario also influences the patient’s perception of the medical service quality. However, most participants complained that the hard equipment of the institution was too old and must be upgraded to improve the quality of the IMS. Participant 10 described our outpatient area as a high-class warehouse by stating:

“There is still a huge gap between us and the top cancer hospitals in the United States. The waiting room in cancer hospitals in the U.S. offers sofas, carpets, and services. We are far behind them. Our waiting area is now a little bit more upgraded than warehouses and is similar to an advanced warehouse.”

Participant 14 even mentioned: “*Because our physicians are strong enough, and our medical equipment and reputation are good enough, patients are willing to come and sit on this broken chair. However, we cannot ignore the equipment and facilities just because our iatrotechnical is good.”*

In addition to the outpatient area, the HCP also strongly criticized inpatient wards. Participant 11 was even shamed for telling others about the ward equipment of the hospital. He stated: *“I am embarrassed to say that our hardware needs to be drastically improved as most of our ward equipment does not even meet the 3-star standard.”* In addition to the limitations of the general hardware, the participants also mentioned that the features of oncology are also different from most surgical divisions. Although the study site offers a specialized ward for IMS, it does not have a specific oncology HCP; thus, it cannot handle the treatment and care issues that may arise in oncology patients. As a result, international cancer patients must be admitted to local cancer wards, which affects the quality of IMS that they receive. For example, Participant 16 stated:

“The more troublesome thing is that the IMS ward cannot administer chemotherapy or perform specialized care because the IMS ward is a service-oriented ward, not a specialist-oriented ward. They cannot even deal with chemotherapy extravasation. We still have to deal with it. In addition, follow-up observation is required after treatment, so professional care is needed. After a patient receives a chemotherapy or immunotherapy drug, even if he or she is fine today, it does not mean that he or she will be fine tomorrow. However, in general surgery, if there are no vital sign changes within 24 hours after the operation, the chance of the patient’s recovery in the next day is much higher.”

Additionally, Participant 18 stated: *“In our current IMS ward, in fact, many treatments cannot be administered. More dangerous and complicated care actually cannot be administered there… our oncology patients cannot go there either.”*

The insufficient number of hospital beds is also a significant issue for the oncology department. Regarding the study site, as it needs to accommodate 20% of the local cancer patients in Taiwan, the number of hospital beds is already very limited. Participants believed that the continuous stream of international patients would definitely exceed the load of the ward and undermine the rights and interests of local patients to seek medical treatment. As a result, many physicians will reject international patients, which correspondingly limits the expansion of IMS. Participant 16 noted:

“It is already difficult for our Taiwanese patients to wait for a bed, and the oncology department is almost one of the worst for this wait. If the hospital separates a section for international patients, it will make Taiwanese patients suffer even more. If the rights and interests of the vast majority of local patients are compromised for the sake of a few special VIPs (meaning international patients), all the physicians will give up the idea of taking care of international patients.”

### Promoting factors

Although IMS has faced several predicaments, participants still provided some suggestions, which had positive effects on promoting the service volume and quality of IMS. The promoting factors included various publicity strategies, one-on-one service types, design of a designated area, and reasonable benefit distribution. Publicity has been most important and indispensable to IMS development. Compared to the official promotional activities occurring in various countries, informal publicity among patients (e.g., social media) has a greater influence on the promotion of IMS, as it allows for more potential customers to understand Taiwan’s medical services and exchange experiences. For example, Participant 13 noted:

“We have been to other countries for formal publicity, which takes place mostly in local hotels. However, informal publicity is more effective than formal publicity. The patients think highly of us and when they return to their homelands, their positive feedback is spread throughout the country very quickly through WeChat.”

He also mentioned: “*The biggest change in Mainland China in recent history is actually the development of social media. The parents of sick children often form a WeChat group wherein they exchange information. This includes not only which hospital they go to receive treatments but also the particular treatments each hospital provides (e.g., proton therapy or photon therapy). Our patient base rises exponentially because of this kind of platform.”*

Additionally, Participant 7 noted that word-of-mouth created strong contributions to IMS promotion by stating: “*When a physician does a good job and successfully treats patients, the word-of-mouth spreads among patients.”*

After the successful promotion of publicity, an increasing number of international patients will be attracted to Taiwan to receive medical services. Most of the participants indicated that international patients who come to Taiwan for medical treatment ask for one-on-one service, wherein a case manager with a nursing background provides exclusive care from the beginning to the end of the treatment, including arranging treatment schedules and assisting patients in communicating with the team. Moreover, a single communication window for case managers can also effectively solve the medical-related issues of international patients and reduce their waiting time, thus decreasing their emotion of helplessness. For example, Participants 7 and 8 stated the following:

“Their demand for service is truly high, and they actually prefer one-on-one full-time care.”

“One-on-one contact makes them feel that they are not coming to the counter and then received by different people every day. They won’t feel that they don’t know the physician or face different windows every day. Patients are able to have someone who can directly help them talk to the team about their needs, make arrangements and then talk to them. Most importantly, patients won’t feel helpless.”

International patients can only stay in Taiwan for a limited amount of time (e.g., approximately one month) due to visa issues; therefore, they require urgent, prioritized treatment. Hospitals should dedicate areas for international patients to reduce waiting times and increase efficiency. For example, Participant 6 noted:

“Our medical team has visited the international medical institutions of other countries, and in comparison, patients may feel that our hard equipment is not as good. Some of them have even complained about why we don’t have a special area for international patients. For example, the outpatient clinics, inspection rooms, and operating rooms are in the same area for the patients’ convenience.”

Most of the participants who were physicians considered that the design of a designated area could avoid affecting the rights and interests of local patients who seek medical treatments, as well as ensuring the quality of international patients. For example, Participant 11 stated:

“If the hospital intends to develop IMS, it should have independently dedicated wards and independent ward nurses who are equipped with foreign language skills. The international wards need to have exclusive care physicians who specialize in medicine and surgery who can take turns at the international wards, and various related departments of medicine and surgery should offer assistance. The most important thing is that such wards cannot occupy the space of the national health insurance wards, or that will be unfair to local patients.”

As mentioned previously, the current reward mechanism of IMS is unfair, and the reward is not proportional to the efforts of the HCPs, which may easily reduce their service enthusiasm. Consequently, most participants suggested that there should be a reasonable distribution of benefits. Specifically, the reward be attributed to both the HCP who actually cares for international patients and to the departments. Only in this way can a hospital effectively enhance the motivation of the team to actively promote IMS and elicits benefits for more international cancer patients. For example, Participant 14 noted:

“The hospital cannot just distribute all the benefits to the HCP who participate in international patient care because other HCP will intend to practice medicine in IMS, too! Moreover, other departments and their HCP will be dissatisfied because their efforts are not rewarded. Therefore, there must be a certain proportion when performing benefit distribution, and the proportion for the actual participants will certainly be higher. However, some benefits must be distributed to the department, while others must be distributed to the hospital. In this way, the government will eventually receive taxes! This would be a wonderful plan!”

He also indicated that 30% of the benefits should be distributed to the department to effectively improve the willingness of HCPs to serve international cancer patients. He stated: *“30% of the benefits must be distributed to the department so that people (HCP) are willing to participate in it (IMS). When the hospital is making money, the HCP is also happy because they know that their efforts will be rewarded. In this way, they are willing to offer services (rapid clinic service) to international patients!”*

## Discussion

Few studies have investigated the experiences and perceptions of IMS quality among HCPs who care for international cancer patients. Four major themes were derived from in-depth one-on-one interviews. In terms of the suggestions on patient selection, psycho-oncological care, predicaments of IMS, and promoting factors for improving IMS care quality, the four major themes were determined to be interrelated, thus emphasizing the difficulties in providing IMS to oncology patients, which is worthy of consideration for hospitals. Each major theme was compared and discussed as follows.

### Patient selection

There was limited previous literature associated with the medical quality of IMS, and most of these few studies were quantitative studies. There were only six qualitative studies [16, 50–52], among which three studies evaluated effective factors affecting IMS from the perspectives of stakeholders and experts of ministries/organizations [51, 53, 54]; in addition, three studies investigated the factors affecting the IMS quality from the perspective of patients and family members receiving IMS [16, 50, 52]. To date, there have not been any studies investigating IMS quality from the perspectives of HCPs, which was also the purpose of this study.

Previous studies associated with the quality of IMS have not mentioned the phenomenon of patient selection; however, the participants in this study repeatedly mentioned the characteristics of patients and disease types that are suitable and unsuitable for IMS. Due to the disease characteristics of rapid progression of advanced cancer and the fact that international patients cannot stay in Taiwan for a long period of time to receive treatment, this study found that some of the physicians attempted to perform patient selection. Furthermore, their attitudes toward participating in IMS is passive and conservative. Even though international cancer patients can be treated, standard treatments (e.g., concurrent chemoradiotherapy) typically cannot be performed due to the lower tolerance of patients to the side effects of treatments. Treatments sometimes have to be discontinued, thus leading to physicians’ failure to make every attempt possible to treat patients. This situation will certainly affect the therapeutic effect and have a negative impact on the morale of HCP and international patients. For hospital managers, at the beginning of promoting IMS, in addition to considering the strengths of in-hospital medical care, it is also necessary to consider the disease characteristics of patients. If hospital managers intend to attract international patients to Taiwan to seek treatment, there is a choice as to how treatments should be determined (e.g., surgical one-time treatment vs. repeated long-term treatment in oncology) for promotion. Taking South Korea and Thailand as examples, these two countries mainly promote plastic surgery and devote themselves to IMS marketing strategies [11, 55]. Under this circumstance, these two countries combine plastic surgery with tourism to enable international patients to visit well-known tourist spots during the trip before the implementation of surgery, which is quite a reasonable scenario. In contrast, the study site is famous for its cutting-edge proton therapy equipment and has attracted many international cancer patients to seek medical treatments. Most of these patients suffer from advanced cancer and are extremely fragile from both physical and mental aspects. It is impractical to combine medical treatments with tourism because such patients are too weak to engage in tourist activities. Therefore, it is inappropriate to adopt such a marketing strategy. Moreover, from the perspective of HCPs providing IMS, patients suffering from advanced cancer may not be suitable for long-distance travel to receive international medical services because the benefits are very limited. All of the abovementioned findings may serve as a reference for relevant decision-makers in hospitals or the government when promoting IMS.

### Psycho-oncological care

Another major theme is associated with psycho-oncological care. Based on the care experiences of HCP, international cancer patients have a strong need for psycho-oncological care, which is certainly associated with disease characteristics. According to previous studies, 20–61% of cancer patients in Europe, America, and Asia will experience emotional distress, and up to 71% of patients with advanced cancer will experience this symptom [56–58]. The analysis of the National Health Insurance Database in Taiwan also found that cancer patients (especially head and neck cancer patients) have 3.8 times more suicide attempts than healthy people because of changes in body image [59]. Therefore, the National Comprehensive Cancer Network (NCCN) has repeatedly emphasized that HCP needs to regularly screen cancer patients for emotional distress and recommends that emotional distress be listed as the sixth vital sign of cancer patients (the fifth vital sign is pain assessment) [56]. Most of the previous studies on IMS enrolled surgical patients who underwent operations as the main subjects [50, 52]. However, these studies never mentioned any findings related to psychological care because most of these international patients returned to their home countries and resumed their normal lifestyles after undergoing one-time medical surgery.

In addition, if the purpose of IMS is to undergo cosmetic surgery, most of these patients seek medical treatment abroad with a sense of joy and anticipation, which is quite different from the mood of international patients in oncology. International cancer patients have to experience painful side effects from treatments, as well as the uncertainty of treatments and even the threat of death. Therefore, it is necessary for HCPs to provide optimal psycho-oncological care. In particular, in the past 3 years, the world has been experiencing the COVID-19 pandemic. Cancer patients have worried about the possible risk of infection when they sought medical treatment and even faced the threat of home isolation or lockdown [60, 61]. Therefore, in the post-pandemic era, the question of how to design a convenient medical visa for international patients and provide safe and fast medical treatment channels, as well as immediate psycho-oncological care, is very important for IMS in oncology.

### Predicaments

Another major issue identified in this study was the IMS predicaments. The site of this study is a well-known medical center in Taiwan, which not only serves 20% of cancer patients in Taiwan but is also equipped with cutting-edge proton therapy equipment and technology. Compared with proton therapy in Europe and the United States, the study site offers both an advantage in terms of price and the cordial service of HCP, which has also received positive feedback from international patients. Therefore, the study site attracts cancer patients from throughout the world for medical treatments. The study site seems to have created huge economic benefits; unfortunately, the predicaments hindering the promotion of IMS were easily observed in this study. In particular, the severe manpower shortage has made HCP experiences miserable. Similar to the findings of the current state of IMS in Hong Kong, the lack of manpower was a problem repeatedly mentioned by every respondent. Heung et al. after interviewing 12 physicians, senior managers, and top executives in charge of international medical departments, found that IMS should be a specialized field but, unfortunately, it lacks experts in nursing and other relative areas [54]. Therefore, manpower at the study site cannot meet the expectations of international patients demanding speedy, high-quality care. Since all services are rooted in manpower, to improve the quality of IMS, lack of manpower must be addressed first and foremost.

Additionally, IMS is a unique industry because it falls under both the medical and service fields, which sets these HCPs apart from general medical personnel. Based on the care experiences of the participants, the care needs of international patients in the oncology department will be higher than those of general international patients (e.g., those patients undergoing surgeries or general health examinations) or local cancer patients. The HCP who offers IMS must not only possess professional medical knowledge and excellent customer service but also have the ability to address the patients’ issues beyond medical care and promptly handle all of the problems during their treatment process. Therefore, under the condition of limited manpower, meeting the tedious and high-standard requirements of international patients will undoubtedly exhaust HCPs. Currently, if there is no reasonable incentive mechanism, it will only reduce the enthusiasm and willingness of HCPs to take care of international patients and subsequently affect the quality of IMS. Therefore, hospitals should not only arrange sufficient HCP manpower but also pay more attention to establishing a fair and motivational reward system. In addition to software issues, limitations in hardware equipment and oncology departments also hinder the study site from promoting oncology IMS. Although the study site has high-end medical technology and cutting-edge treatment equipment and has passed the International Joint Commission International (JCI) accreditation certification, the core belief of this medical institution is “diligence and simplicity”. For usable hardware equipment, especially equipment in inpatient wards, the study site rarely makes updates, even if such equipment is old and outdated. Local patients may be accustomed to this phenomenon. However, for international patients from high socioeconomic classes who require long-term hospitalization, this phenomenon will inevitably affect their trust in hospitals and their appraisal of IMS quality. This scenario differs from a previous qualitative study that interviewed 18 Chinese medical tourists on their experiences of Taiwan’s medical services and discovered that patients were satisfied with the five-star service of Taiwanese hospitals and especially felt comfortable in the inspection area that distinguishes local patients from healthy medical tourists [16]. However, more than half of the 18 Chinese patients only received health check-ups or used facilities that were mostly concentrated in the outpatient area [16]. The duration of their medical procedure was also shorter in comparison to patients with advanced cancer; thus, their experiences and expectations may be different. The treatment of cancer patients occurs for a longer period of time and often requires hospitalization, which may lead to higher expectations from the hospital facilities. Previous studies have suggested that high-end medical equipment is the key to promoting IMS quality [13, 55]. If hard equipment cannot regularly be updated and maintained, IMS development will definitely be hampered.

Moreover, due to the unique characteristics of oncology care, although the study site offers luxuriously designed IMS exclusive wards, the wards can only provide general care and cannot provide specific oncology care. Therefore, if international cancer patients need to be hospitalized, they cannot live in a luxurious IMS ward but instead need to live in the general oncology ward. The equipment and level of comfort of the oncology ward are definitively not as good as those of a luxurious IMS ward. Additionally, when international patients are admitted to the oncology ward, they will inevitably compete with local cancer patients for care resources. Moreover, in the absence of improvements in manpower allocation and incentive measures in hospitals, the workload of HCPs in the oncology department will continue to increase, thus leading to a vicious cycle of IMS in the oncology department. British scholars have also indicated that the development of IMS will indeed reduce the medical care received by local patients. Therefore, they emphasize that when developing IMS, exporting countries (those countries that provide health services to international patients) need to develop a better regulatory framework to guide the provision of services to international patients and adopt equitable buying guidelines to address the impacts of IMS on equity. This regulatory framework can ensure the service boundary of HCP and the service quality for international patients while also protecting the medical rights and interests of local patients [62, 63].

### Promoting factors

To avoid the abovementioned predicaments and increase the quality of IMS, the participants also provided improvement suggestions based on their own experiences. These suggestions are expected to provide a reference direction for hospitals that intend to develop IMS or that have developed IMS but are experiencing bottleneck issues. According to the in-hospital statistics of the study site, more than 80% of international patients are from Asia, and other patients originate from Europe, America, or the Middle East. Apparently, IMS in Taiwan still has a significant need for promotion in non-Asian countries. Many HCPs indicated the ability of formal publicity to significantly boost IMS development, and previous studies have similar findings [23, 64–66]. Previous studies have also found that expanding the channels of publicity, building brand image, and providing customers with the necessary information is the best marketing strategy to promote IMS [40, 67, 68]. A systematic review also indicated that advertisements, friend recommendations, good reputations, and brand trust affected patients’ motivations in IMS [40].

Additionally, given the current popularity of social media, the participants have repeatedly emphasized the immediacy and effectiveness of internet dissemination of information. As long as the hospital has cutting-edge medical technology and good service quality, the good reputation of the hospital’s IMS will be disseminated through the internet, social media, or word-of-mouth among patients. Moreover, it will attract more foreign patients to seek IMS [27]. Previous studies have also demonstrated that the sharing of experiences among patients can considerably boost the promotion of IMS development [64]. Most international cancer patients who come to Taiwan have high expectations for our IMS. Moreover, due to the high medical fees and their preference for privileged care, most of these patients would prefer to have one-on-one dedicated services. However, the current manpower situation of the study site cannot meet the expectations of these patients. If the hospital can improve the shortage of manpower, every international patient who plans to come to Taiwan will have a specific HCP (e.g., case manager) to assist in the schedule arrangement before coming to Taiwan, as well as assist in the treatment process upon arrival in the hospital and the post-treatment follow-up after returning to their country. This circumstance will greatly improve IMS quality and international reputations in Taiwan. Previous studies have also found that international patients prefer to receive all services in a one-stop/stage manner so that they can obtain personal attention and rapid service [40], which is consistent with the one-on-one specific services suggested by the participants of this study.

Moreover, the participants were worried that international cancer patients would affect the rights and interests to medical treatment of local cancer patients and that the hospital would request HCPs to take care of international and local cancer patients at the same time, which would undoubtedly worsen this situation. Therefore, the participants suggested the design of an exclusive area for IMS and the arrangement of exclusive care HCPs, which can not only guarantee the quality of treatment for international patients but also avoid compressing the rights and interests of local patients. Additionally, a “long waiting time” was a common complaint among international patients [52]. Most of the HCPs believe that designing dedicated areas can effectively improve service quality and increase patient satisfaction. The Medical Tourism Association (MTA) indicated that the efficient facilitation of patients’ medical treatments will effectively improve service quality [64]. Moreover, previous studies have found that patients opt for medical treatments abroad because they must wait for a long period of time to access treatment in their home countries [52, 64]. In particular, patients with advanced-stage cancers have greater urgency; thus, the earlier they can access the treatment, the better the outcomes could potentially be.

Currently, although many Asian countries have hospitals dedicated to serving international patients (such as Singapore, India, Thailand, and South Korea), most of them provide medical services related to surgical treatments (including cosmetic/plastic, dental, cardiac, or bariatric surgeries) or fertility treatments [69–71]. Given that cancer has become one of the most common diseases with a high mortality rate throughout the world, the demand for oncology IMS will increase. Therefore, the IMS services and related studies for such patients should match this demand, which is also a neglected point of previous IMS research.

Last, the participants mentioned a sensitive issue: although the study site has provided bonuses to HCPs who care for international cancer patients, the bonus amount is so small that HCPs believe that it is better to save precious time for local cancer patients than to receive meager rewards. Some HCPs even mentioned the issue of “medical equality”. The importance of medical equality has been constantly emphasized in medical and nursing education in Taiwan; therefore, they believe that medical treatment should be a medical profession rather than a service industry. HCPs must do their best to help patients regain their health. However, the current IMS promoted by hospitals obviously includes the medical and service levels, which creates contradictions in the core value of HCPs and makes HCPs doubt whether medical care is a service industry and how IMS should be positioned. Previous studies have emphasized that hospital managers and government authorities need to establish their positioning for IMS, which is conducive to the marketing and development of IMS [68]. Moreover, there is a question as to if medical service is a medical profession or a service industry, as well as if IMS is different from traditional medical care. These core value issues are worthy of reflection by industry, government, and academia. Moreover, some IMS studies have found that language communication and cultural customs have an impact on IMS quality; however, this study did not observe an impact of the abovementioned factors on service quality. This phenomenon may have been related to the fact that more than 80% of the international patients in the study site are Chinese individuals from Asian countries. HCPs can use Mandarin to communicate with these international patients, and these patients are very similar to Taiwanese people in terms of their culture, values, beliefs, and living habits.

### Limitations and strengths

Most IMS studies have focused on developing strategies to attract international patients for economic benefits [8, 11, 72]. This is the first study focused on the perceptions of HCPs who care for international cancer patients. However, this study had some limitations. First, our study site is a medical center in northern Taiwan. Therefore, the findings of this study may not be generalizable to IMS in all of Taiwan. Second, most of our respondents were doctors and nurses who had close contact with international patients. However, during the treatment process, the radiologist interacted quite often with the patients, especially with those patients who were receiving proton therapy. Thus, our results may not be transferable to other HCPs. Third, our study focused on the perspectives of HCPs. However, the opinion of patients is also valuable for improving the IMS. Consequently, we conducted further research that explored patients’ perspectives. Fourth, different ethnicities and races of HCPs may have different experiences in caring for international cancer patients. However, all of the HCPs caring for international patients were Taiwanese and were interviewed in Mandarin, which may have limited the transferability of this study.

Although the global health crisis caused by COVID-19 has significantly affected IMS development [73], we view this situation as an opportunity for hospitals to establish and fortify their IMS, and the findings from this study could inform high-quality IMS development both during and after the pandemic. Hospital administrators are advised to seize the opportunity to make substantial improvements to IMS quality. Additionally, international patients are mostly people of high social standing who are financially capable of seeking the latest, fastest, and most high-end medical care abroad at their own expense. Although IMS can be very profitable for the hospital and the country overall, it may cause a crowding-out effect on local patients [74]. The increasing number of international patients may infringe on the rights of local Taiwanese patients. Therefore, it is recommended that hospitals carefully develop the necessary hardware and software to provide the highest-quality IMS to patients from any part of the world [74].

## Conclusions

This study aimed to explore **oncology** HCPs’ experiences of IMS quality in caring for international cancer patients, with implications for hospitals in developing high-quality IMS. During the data analysis process, four major themes emerged: selecting appropriate patients, paying attention to psycho-oncology care, managing several predicaments, and taking the promoting suggestions into consideration. Given that IMS is a global trend, the findings from this study provide new insights for HCPs, administrators, and policy-makers in improving the quality of IMS in the oncology department, which was the least studied field in IMS quality. It also offers possible implications for future researchers to compare the perceptions and experiences among different populations (e.g., HCPs, patients, and stakeholders) to further enhance our understanding of IMS quality from various viewpoints.

## Data Availability

The datasets generated and/or analyzed during the current study are not publicly available due to ethical issues (e.g., due to the fact that they contain information that could compromise the privacy of research participants) but are available from the corresponding author on reasonable request.

## Abbreviations

IMS: International Medical sSrvice
WHO: World Health Organization
WTO: World Tourism Organization
HCP: Healthcare personnel
MTA: Medical Tourism Association.
